# The Role of Coping in the Wellbeing and Work-Related Quality of Life of UK Health and Social Care Workers during COVID-19

**DOI:** 10.3390/ijerph18020815

**Published:** 2021-01-19

**Authors:** Paula McFadden, Jana Ross, John Moriarty, John Mallett, Heike Schroder, Jermaine Ravalier, Jill Manthorpe, Denise Currie, Jaclyn Harron, Patricia Gillen

**Affiliations:** 1 School of Applied Social and Policy Sciences, Magee Campus, Ulster University, Londonderry BT48 7JL, UK; j.ross@ulster.ac.uk; 2 School of Social Sciences, Education and Social Work, Queen’s University Belfast, 69-71 University Street, Belfast BT7 1HL, UK; j.moriarty@qub.ac.uk; 3 School of Psychology, Coleraine Campus, Ulster University, Cromore Road, Coleraine BT52 1SA, UK; j.mallett@ulster.ac.uk; 4 Queen’s Management School, Queen’s University Belfast, Riddel Hall, 185 Stranmillis Road, Belfast BT9 5EE, UK; h.schroder@qub.ac.uk (H.S.); d.currie@qub.ac.uk (D.C.); 5 School of Science, Bath Spa University, Newton Park, Newton St Loe, Bath BA2 9BN, UK; j.ravalier@bathspa.ac.uk; 6 NIHR Health and Social Care Workforce Research Unit, King’s College London, London, 22 Kingsway, Holborn, London WC2B 6LE, UK; jill.manthorpe@kcl.ac.uk; 7 Independent Researcher, Derry BT48 8RS, UK; jackiemharron@gmail.com; 8 School of Nursing, Jordanstown Campus, Ulster University, Shore Road, Newtownabbey BT37 0QB, UK; p.gillen@ulster.ac.uk or; 9 Southern Health and Social Care Trust, 10 Moyallen Road, Gilford BT63 5JX, UK

**Keywords:** health and social care, coping, quality of working life, wellbeing, COVID-19

## Abstract

The coronavirus disease 2019 (COVID-19) was declared a global pandemic in early 2020. Due to the rapid spread of the virus and limited availability of effective treatments, health and social care systems worldwide quickly became overwhelmed. Such stressful circumstances are likely to have negative impacts on health and social care workers’ wellbeing. The current study examined the relationship between coping strategies and wellbeing and quality of working life in nurses, midwives, allied health professionals, social care workers and social workers who worked in health and social care in the UK during its first wave of COVID-19. Data were collected using an anonymous online survey (*N* = 3425), and regression analyses were used to examine the associations of coping strategies and demographic characteristics with staff wellbeing and quality of working life. The results showed that positive coping strategies, particularly active coping and help-seeking, were associated with higher wellbeing and better quality of working life. Negative coping strategies, such as avoidance, were risk factors for low wellbeing and worse quality of working life. The results point to the importance of organizational and management support during stressful times, which could include psycho-education and training about active coping and might take the form of workshops designed to equip staff with better coping skills.

## 1. Introduction

Coronavirus disease 2019 (COVID-19), which was declared a global pandemic by the World Health Organization on 11 March 2020, placed substantial strain on health and social care systems worldwide. The rapid spread of the virus, compounded by a limited range of effective treatments, meant that health and care systems struggled to cope [1]. However, it quickly became evident that the negative impact of COVID-19 was felt not just at the organizational level, but also at the individual level, as studies of the mental health and wellbeing of health and social care staff started emerging. Providing care and treatment during a pandemic can be associated with fear, worry and anxiety [2]. It may be accompanied by anger and frustration resulting from the rapidly evolving working conditions which are often sub-optimal due to the general unpreparedness of some health and care systems (e.g., lack of personal protective equipment (PPE) [3]). Staff may worry about contracting the virus at work and bringing it home. They may be redeployed to new roles for which they feel unprepared, their workloads increase, or they may feel anxious about their organization’s support should they become ill [4].

Emerging studies have documented the negative effects of working at the frontline in health and social care during COVID-19. To date, the vast majority of these have been conducted in China [5], where the virus is thought to have originated. For example, Lai et al. [6] examined the mental health of 1257 staff in 34 hospitals in China and found large proportions reported symptoms of depression (50.4%), anxiety (44.6%), insomnia (34.0%) and distress (71.5%). A study of 1153 Italian healthcare professionals found high levels of emotional exhaustion (significantly higher than in a normative sample) and frequent somatic symptoms [7]. Another Italian study found healthcare professionals working in COVID-19 wards reported higher levels of depression and posttraumatic stress symptoms compared to those working elsewhere [8]. These are unsurprising findings, in line with other studies of the impact of viral epidemic outbreaks on the mental health of healthcare workers [9].

Healthcare staff are a high-risk group when it comes to stress, burnout, and lower levels of wellbeing, even during non-pandemic times. In the UK, this is generally a result of increasing work-related demands, decreasing staffing levels, and other diminishing resources within the healthcare system [10]. In 2018, the state of the UK healthcare system, the National Health Service (NHS), was described as unsustainable by the General Medical Council [11]. A 2018 NHS Staff Survey revealed that 43.5% of nurses and midwives in England reported being unwell due to work-related stress [12]. Levels of burnout in nurses and midwives have been reported to be higher in the UK than in other countries, and UK nurses and midwives also report higher levels of mental health problems, such as depression and anxiety, compared to the general working population [12,13].

Other health and social care occupations in the UK, such as social workers, were also reported to be experiencing high levels of work-related stress prior to the outbreak of COVID-19 [14,15]. Research on social care workers’ or support workers’ wellbeing in the UK is limited, but suggests that factors such as low pay, insecure ‘zero-hour’ contracts, and poor work–life balance are significant stressors in this workforce [16]. It could be argued that the added pressures of COVID-19 would put the health and social care workforce under even more strain and lead to more stress and potentially lower wellbeing.

Yet, there are differences between individuals in how they deal with the increased work-related demands and stressful situations. Stress is the interaction between an individual and their environment. A disparity between the perceived demands of a situation and an individual’s perceived ability to cope with these demands will result in the person feeling stressed if the demands are perceived to be high and the ability to cope is perceived to be low [17]. The coping process therefore consists of two parts; the primary appraisal of the event as being harmful or threatening, and the secondary appraisal of one’s own coping options or mechanisms that can be used to deal with the potentially stressful event or situation [18,19]. Research has demonstrated individual differences in coping with stress (e.g., [20,21,22]) as well as the relationship of different coping strategies with wellbeing and work-related quality of life. In relation to the latter, a systematic review of 17 studies conducted with emergency nurses revealed that positive coping strategies, such as active problem-focused coping, were associated with lower levels of burnout, whereas negative coping strategies, such as passive avoidant coping, were ineffective when dealing with stress [23]. Other studies have reported similar relationships between coping strategies and levels of wellbeing, including the quality of working life, both in nurses and in other health and social care professionals [24,25,26,27,28,29].

### 1.1. Theoretical Framework

The current study could be situated within the Job Demands Resources model, which postulates that, within any occupation, there are specific job demands and job resources, and the interaction of these determines whether job stress or other outcomes, such as burnout or low wellbeing, will be experienced. Job demands are aspects of the job that require sustained physical or mental effort and may therefore lead to the above negative outcomes. On the other hand, job resources are those aspects of the job that reduce the job demands, help one achieve work goals and stimulate personal growth [30]. More recently, the role of personal resources has been incorporated into the model, as psychological characteristics of one’s self that are associated with resiliency. These personal resources may help explain the individual differences in people’s wellbeing despite them being exposed to the same job demands and resources [31]. The COVID-19 pandemic has undoubtedly increased the job demands facing the health and social care workforce, which, as the emerging research suggests, have led to negative impacts on staff’s wellbeing (e.g., [6]). Not everyone will, however, be affected in the same way, and this is where personal resources, such as one’s coping strategies, could play an important role.

### 1.2. Aims of the Study

The current study aimed to examine the relationship between coping strategies, wellbeing and the quality of working life in nurses, midwives, allied health professionals (AHPs), social care workers, and social workers who were working in health and social care in the UK during its first wave of the COVID-19 pandemic (May–July 2020). It is important to examine these issues, because with an increasing influx of patients and decreasing staffing levels due to sickness, health and care systems worldwide have become stretched [32]. Staff sickness absence can be due to physical illness, but also due to stress, burnout, or other problems. Additionally, research has suggested that stress at work and burnout can lead to lower quality of care [33,34,35]. The current study considered different coping strategies and demographic characteristics as potential risk (demands) and protective factors (resources) for wellbeing and work-related quality of life. As such, the results of this study have implications for supporting the health and social care workforce during other periods of system stress or future viral outbreaks or pandemics. It was hypothesized that positive coping strategy resources will be associated with higher wellbeing and better quality of working life, and negative coping strategies will be associated with lower wellbeing and worse quality of working life by adding to demands experienced.

## 2. Materials and Methods

### 2.1. Data and Participants

The data for this study come from an ongoing program of research entitled ‘Health and social care workers’ quality of working life and coping while working during the COVID-19 pandemic’, which was launched in May 2020 with the first in a series of three cross-sectional surveys. The research program aims to explore the impact of providing health and social care during the COVID-19 pandemic on nurses, midwives, AHPs, social care workers, and social workers. The current study is based on data collected through the first self-report survey, which was available online between 7th May and 3rd July 2020. The survey was available in all four devolved regions of the UK: England, Wales, Scotland, and Northern Ireland and received 3425 responses. Respondents were recruited through emails, newsletters, and social media posts of professional regulatory bodies, associations, and workplace unions for nurses, midwives, AHPs, social care workers, and social workers.

### 2.2. Measures

The online survey contained questions on demographic information, validated scales assessing respondents’ wellbeing, work-related quality of life, and coping strategies and also open-ended questions (not relevant to the current paper) enquiring about their experiences of working during the early part of the COVID-19 pandemic in the UK.

Demographic information relevant to the current paper included sex, age, ethnicity, relationship status, respondents’ current place of work (England, Scotland, Wales, Northern Ireland), occupational group (nursing, midwifery, AHP, social care, social work), whether they had a disability, number of sick days taken over the previous year, and whether they had been re-deployed due to the pandemic.

Respondents’ wellbeing was assessed using the seven-item self-report *Short Warwick Edinburgh Mental Wellbeing Scale* (SWEMWBS [36]). Using a five-point Likert scale (1 = ‘None of the time’ to 5 = ‘All of the time’), respondents were asked to indicate how often over the last two weeks they had feelings or thoughts described in the statements (e.g., I’ve been feeling useful). The scores for the seven items were summed and transformed into metric scores [36], ranging from 7 to 35. Higher scores indicate better mental wellbeing. The scale had good internal consistency in the current study (Cronbach’s alpha = 0.86).

The *Work-Related Quality of Life* (WRQOL) scale [37] assessed respondents’ quality of working life. Respondents were presented with 24 work-related items (e.g., I have a clear set of goals and aims to enable me to do my job) and were asked to indicate their agreement with these using a five-point Likert scale (1 = ‘Strongly disagree’ to 5 = ‘Strongly agree’). The scale measures six different domains, specifically, Control at work, General wellbeing, Home–work interface, Job career satisfaction, Stress at work, and Working conditions. In the current study, 23 items, which contribute to the final score, were totaled (as per the WRQOL manual), with possible scores ranging from 23 to 115. Higher scores indicate better WRQOL. Cronbach’s alpha for the 23 items was 0.88, suggesting good internal consistency.

Coping with COVID-19-related occupational demands was assessed by two different scales. Firstly, respondents completed the 28-item *Brief COPE* scale [38]. The items represent 14 different coping strategies and respondents indicate how often they have been using them on a four-point Likert scale (1 = ‘I haven’t been doing this at all’ to 4 = ‘I’ve been doing this a lot’). We used the 28 items in an exploratory factor analysis to generate a smaller number of factors to use as predictors in the regression analysis (to reduce the number of predictors). The mean score for each factor was calculated from the items within the factor. Higher scores indicate that respondents use a specific coping strategy more frequently. Internal consistency of the 28 items was good in the current study (Cronbach’s alpha = 0.82).

Secondly, respondents completed 15 items from Clark, Michel, Early, and Baltes’ scale [39]. The 15 items represent five coping strategies (Family–work segmentation, Work-family segmentation, Working to improve skills, Recreation and relaxation, Exercise) which were selected out of Clark et al.’s 12 work stressor coping strategies to supplement those assessed by the Brief COPE scale. Respondents were asked to indicate how often they have done what was described (e.g., Actively try to keep my work problems at work) to cope with work stressors, using a six-point Likert scale (1 = ‘Never have done this’ to 6 = ‘Almost always do this’). Each coping strategy is represented by three items, and a mean score calculated for each coping strategy. Higher scores indicate increased frequency of using a specific coping strategy. The Cronbach’s alpha of the 15 items was 0.83, indicating good internal consistency.

### 2.3. Data Analysis

We first conducted an exploratory factor analysis (EFA) with principal axis factoring to derive a smaller number of coping strategies from the 28-item Brief COPE scale. We interpreted the solution after a varimax rotation (oblimin rotation produced comparable results).

We then conducted two forced entry multiple regressions; one with the wellbeing scores as an outcome and one with the WRQOL scores as an outcome. The following variables were used as predictors in both models: Brief COPE domains derived from EFA (mean scores), Clark et al.’s coping strategies (mean scores), and dummy-coded demographic variables of sex (female, male, [‘neither’ was treated as missing due to low sample size: n = 8]), age (16–29, 30–49, 50–65, 66+), ethnicity (white, mixed, black, Asian), relationship status (married, single, divorced/separated, cohabiting), place of work (England, Scotland, Wales, Northern Ireland), occupation (nursing, midwifery, AHP, social care, social work), disability (no, yes, unsure), number of sick days (none, less than 10, 11–20, 21–60, more than 60) and redeployment (no, yes). The first category in the bracket represents the reference category. All analyses were conducted in SPSS 26 (IBM, Armonk, NY, USA).

### 2.4. Missing Data

Of the initial sample of 3425 respondents, we excluded 607 who did not complete any questions past the initial demographics section. A further 272 were excluded due to having more than 30% of missing values on the relevant measures, as the validity of this data is doubtful with such high levels of missingness. Additionally, we excluded five respondents who did not state their occupational group, because being a nurse, midwife, AHP, social care worker, or social worker was an inclusion criterion in the current study. This left an effective sample size of 2541 respondents. We estimated the remaining missing data (0.25% values) on SWEMWBS, WRQOL, Brief COPE, and Clark et al.’s coping items using the expectation maximization algorithm. Missing demographic information (0.18% values) was not estimated and listwise deletion (*n* = 36) was employed in the regression analysis.

There were some significant differences between the included (*n* = 2541) and excluded (*n* = 884) respondents. Based on chi-square tests and the examination of adjusted standardized residuals, those who were excluded were more likely to be in the 16–29 age category and less likely to be in the 50–65 age category; they were less likely to be working in England and more likely to be working in Northern Ireland; they were more likely to be social care workers and less likely to be social workers; and they were more likely to be single and less likely to be married. There were no differences between the two groups in sex, ethnicity, disability, number of sick days, or redeployment status.

## 3. Results

### 3.1. Descriptive Statistics

Table 1 shows respondents’ demographic information. The mean mental wellbeing score in the effective sample was 21.35 (SD = 3.58). The mean total WRQOL score was 78.15, which translates to average quality of working life [40]. A total of 792 (31.17%) respondents had low quality of working life, 668 (26.29%) had average quality of working life, and 1081 (42.54%) had high quality of working life. In relation to coping strategies, the most frequently used strategy, out of the 14 Brief COPE strategies, was acceptance (M = 6.49, SD = 1.43), and the least frequently used one was behavioral disengagement (M = 2.62, SD = 1.18). Out of the five Clark et al.’s coping strategies, the most frequently used one was family–work segmentation (M = 5.05, SD = 0.91).

### 3.2. Exploratory Factor Analysis

In the initial exploratory factor analysis of the 28 Brief COPE items, six factors had eigenvalues greater than one, accounting for 53.64% of variance. An examination of the scree plot indicated that the biggest change in slope occurred after three factors. We therefore examined solutions between two and six factors. The two- and three-factor solutions had several items with low factor loadings and factors were difficult to interpret. The four-, five-, and six-factor solutions were easier to interpret. The six-factor solution was selected as it was the easiest one to interpret, with all but two items loading highly (above 0.4) on their respective factors. The two low-loading items (below 0.3) were excluded. The final six-factor solution (26 items) explained 55.07% of variance. We re-ran the factor analysis with oblimin rotation, which yielded the same solution, suggesting that the resulting factors are uncorrelated. Factor loadings for all items are shown in Table 2. The factors were interpreted as Active coping, Avoidance coping, Help seeking, Religion, Substance use, and Humor. 

### 3.3. Multiple Regressions

Table 3 shows the correlations between continuous study variables. Regression model 1, in which the wellbeing scores were the outcome variable, explained 36.0% of the variance (adjusted R^2^ = 0.351, F (35, 2469) = 39.63, *p* < 0.001). Table 4 shows the regression coefficients for all continuous and dummy-coded predictor variables. Briefly, higher scores on active coping, help seeking, religion, humor, work-family segmentation, working to improve skills, recreation, and relaxation and exercise, and lower scores on avoidance coping and substance use all predicted higher wellbeing scores. Additionally, males, those aged 50–65 years, and those working in Northern Ireland had higher wellbeing scores (compared to the reference categories). Those who reported being single or divorced/separated, those working as AHPs or social workers, and those reporting any number of sick days over the previous year had lower wellbeing scores (compared to the reference categories).

Model 2, in which the WRQOL scores were the outcome variable, explained 26.8% of the variance (adjusted R^2^ = 0.257, F (35, 2469) = 25.78, *p* < 0.001). Regression coefficients for all predictor variables are presented in Table 4. Higher scores on active coping, help seeking, humor, work-family segmentation, working to improve skills, and recreation and relaxation and lower scores on avoidance coping and family–work segmentation all predicted higher WRQOL scores. Respondents who reported their ethnicity as black, those who were single or cohabiting, those working in Scotland or Northern Ireland, those with a disability (or those unsure of whether they had a disability), those reporting 11+ sick days in the previous year, and those who had been redeployed due to the pandemic had lower WRQOL scores. The strongest predictor in both models was avoidance coping, which was negatively associated with the outcomes.

## 4. Discussion

The current study examined coping strategies and demographic characteristics as risk and protective factors of the wellbeing and quality of working life in nurses, midwives, AHPs, social care workers, and social workers working in health and social care in the UK during the first months of the COVID-19 pandemic. As hypothesized, positive coping strategies, particularly active coping and help-seeking, were associated with higher wellbeing and better quality of working life. On the other hand, more negative coping strategies, such as avoidance coping, were associated with lower wellbeing and worse quality of working life. Substance use was also significantly associated with lower wellbeing, but not the quality of working life, although the relationship was in the predicted direction.

The most frequently used coping strategy in our sample, out of the 14 Brief COPE domains, was acceptance. Considering the circumstances surrounding the COVID-19 pandemic, the fact that people’s lives, both personal and professional, changed drastically almost overnight, accepting the reality of what has happened and trying to learn to live with it may be the optimal approach. Indeed, research has shown that when coping with situations where perceived controllability is low, people often rely on acceptance [41]. Under specific circumstances, acceptance can therefore be an adaptive coping strategy as it can provide people with closure and allow them to move on in life. If the situation is perceived as unchangeable, acceptance aims to change the emotional reaction to it [42]. In our factor analysis, the two Brief COPE acceptance items loaded on the active coping factor, which was positively associated with higher wellbeing and better WRQOL, and this is in line with the findings of Nakamura and Orth [42], who showed that active acceptance is an adaptive coping strategy, and it is different from resigning acceptance, which is a maladaptive coping strategy.

An important finding of the current study was that the mean wellbeing score, as measured by SWEMWBS, was 21.35, which is over two points lower than the population average (23.7 for men and 23.6 for women) derived from a nationally representative sample of adults living in private households in England [43]. This suggests that the wellbeing of our sample is lower than that of the general population. One explanation for this discrepancy could be the fact that the population average was based on data collected pre-COVID-19 and the pandemic could have contributed to deterioration in wellbeing in both health and care workers and the general population. However, health and social care workers generally report lower wellbeing than the general population. For example, a recent systematic review of 50 studies conducted during the COVID-19 pandemic showed that this population group has higher prevalence rate of psychological morbidities than the general population [5] and similar findings have been reported in pre-pandemic times [12].

In terms of the WRQOL, the mean total score in our sample was 78.15, which indicates that, on average, our sample reported having an average quality of working life [40]. This is comparable to the mean normative score of 3.44 (78.15/23 questions = 3.39) derived from the UK NHS workforce [40]. Interestingly, the normative score was based on data collected prior to the COVID-19 pandemic. This could mean that respondents’ WRQOL was not substantially impacted by the changed working conditions, but longitudinal data would be needed to corroborate this assumption as the two samples are not directly comparable and it is likely that some health and social care occupations and those working in particular settings (e.g., Intensive Care Units (ICUs)) or with particular groups of patients or service users (e.g., children or older people) were affected more than others.

In relation to the results of the multiple regression analyses, we found several significant predictors, which could either be considered risk or protective factors for wellbeing and WRQOL. Avoidance coping, which is generally considered to be a maladaptive coping strategy, as it tends to lead to negative outcomes, was the strongest predictor of both wellbeing and WRQOL; individuals who employed avoidance coping more frequently had lower wellbeing and WRQOL scores. Avoidance coping has previously been identified as one of the coping strategies employed by health and social care workers (e.g., [44,45]), and in our sample, it had the strongest impact on the outcomes measured, suggesting that it may be an appropriate target for interventions, such as psychoeducation and training in positive coping methods. For example, research on coping with work-related stress during COVID-19 points to risks of downstream health problems due to unhealthy ‘comfort’ eating and poor nutritional choices, which may be viewed as another example of avoidance coping that may need addressing [46]. Furthermore, other COVID-19 healthcare worker research [47] has found that the risk of developing Post-Traumatic Stress Disorder was associated with negative coping, and the authors recommended psychological counselling for all frontline healthcare workers. Learning about work-related stress and coping from the COVID-19 pandemic could be adopted into training the workforce for both ‘service as usual’ related coping, as well as for future disasters or pandemics.

Of the more adaptive coping strategies, active coping and help-seeking were found to be protective factors in our sample, as they were associated with both higher wellbeing and better WRQOL. This finding is in line with other research suggesting that active or approach coping strategies are associated with better outcomes (e.g., [23,29]). Work–family segmentation (i.e., not dealing with work-related problems at home), working to improve skills/efficiency and recreation and relaxation were also protective factors for one’s wellbeing and WRQOL in our study. Exercise was associated with better wellbeing and family–work segmentation (i.e., not dealing with family-related problems at work) was associated with lower WRQOL. The latter finding is interesting as it suggests that those who were coping with work-related stressors by actively trying not to deal with their family problems whilst at work had worse WRQOL than those who allowed themselves to think and deal with family problems whilst at work. Common sense might suggest that better segmentation between one’s family and work lives would lead to better balance between the two and less work–family and family–work conflict [48]. More recent research, however, suggests, that this relationship is not so straightforward, as the right balance in the integration of work and family can be associated with family–work or work–family enrichment [49], where experiences in one role enhance the quality of life in the other role [50]. Further research is, however, needed to better understand the effects of family–work and work–family segmentation on wellbeing and quality of working life.

In terms of the Job Demands Resources model, the results suggest that several coping strategies used by staff served as resources (i.e., buffers) protecting them against lower wellbeing and worse quality of working life. Specifically, active coping, help seeking, religion, humor, work–family segmentation, working to improve skills, recreation and relaxation, and exercise were all coping strategies employed by staff (personal job resources) that protected them against low wellbeing. On the other hand, avoidance coping and substance use (personal job demands) were adding to one’s stress, leading to lower wellbeing. In terms of WRQOL, active coping, help seeking, humor, work–family segmentation, working to improve skills, and recreation and relaxation served as personal job resources, contributing to better WRQOL. Avoidance coping and family–work segmentation served as personal job demands and were associated with lower WRQOL.

The results of the demographic characteristics as predictors of wellbeing and WRQOL revealed that having taken sickness leave in the previous 12 months was a risk factor for lower wellbeing and lower WRQOL during COVID-19. Having a disability was also a risk factor for lower WRQOL, but it was not significantly associated with wellbeing scores. Males reported higher wellbeing than females, but no gender differences were found for WRQOL. Emerging studies suggest that COVID-19 has more negative impact on the psychological health of females than males [51,52,53]. It is possible that this is related to the differences in the amount of domestic and childcare work, which has been found to be more often undertaken by mothers than fathers during COVID-19 [54].

Several other demographic variables were significantly associated with wellbeing and WRQOL. Being single or divorced/separated was a risk factor for lower wellbeing, perhaps due to these respondents experiencing more loneliness and/or having less social support [52]. Respondents with black ethnic background were found to have lower WRQOL than those reporting white ethnicity, and this finding is in line with previous healthcare research on the quality of working life of ethnic minority groups [55,56] and highlights the importance of promoting equality and inclusion through employer and managerial support. The proportion of ethnic minority groups in our sample was small, but future studies could focus specifically on these to explore in more detail their WRQOL and factors affecting it. Our results also showed that older respondents (aged 50–65) had higher wellbeing than those in the youngest category (16–29). This could possibly be related to their years of experience and thus better ability to deal with work-related stress. Also important to note is the finding that respondents who had been redeployed to other roles due to the pandemic had lower WRQOL. This is likely related to the fact that of those who had been re-deployed, only 23.46% felt well prepared for their redeployment, whereas 36.59% felt unprepared and a further 39.94% felt neither prepared nor unprepared.

### 4.1. Limitations

The current study has several limitations. Firstly, data were collected online using a self-report survey, which could have been accessed by anyone who had seen the advertisement for the study. It was a requirement of the study that participants were working as nurses, midwives, AHPs, social care workers, and social workers, but the occupations were self-reported. However, since the study was advertised through professional regulatory bodies, associations, and workplace unions for the above occupational groups and no incentives were offered to respondents for completing the survey, it is unlikely that respondents worked in other occupations. Secondly, most respondents were social workers and social care workers, which was probably a result of effective advertising to these occupational groups and the presence of other workforce surveys in the NHS, some of which were directed at nurses and midwives. Additionally, the number of respondents from Scotland and Wales was relatively small, which makes the findings less generalizable to the health and care workers in these countries. The results may also not be fully reflective of the wellbeing and WRQOL of certain healthcare professionals, such as nurses working in extremely intense environments, who may not have had the time to complete the survey during the pandemic. Thirdly, the sample was predominantly female, thus generalizations to male health and care workers should be made with caution. The gender composition of our sample is, however, largely reflective of the wider UK nursing, midwifery, social care, and social work workforce [57,58,59] and also of other countries’ health and social care workforce, which is mainly female, with lower pay and working conditions [60]. Another or accompanying explanation for the gender composition of respondents is also possible; females may have been more motivated to complete the survey, as recent research has shown that males are more interested in extrinsic motivations when participating in life sciences research, whereas females also value intrinsic motivations, such as helping society [61]. Finally, our survey did not assess medical or mental health conditions or any treatments that participants were undergoing (e.g., depression and antidepressant medication), which could have impacted upon the wellbeing and WRQOL scores in our sample. Future studies could control for these variables to improve the robustness of the results.

### 4.2. Implications

Despite their limitations, the results of this study have several important implications. First, avoidance coping emerged as the strongest predictor of both wellbeing and WRQOL. Considering its negative effects on wellbeing and WRQOL, avoidance coping may be a good target for interventions, where health and care workers are helped to replace it with more adaptive coping strategies. Studies have shown that interventions can be designed to increase individuals’ use of adaptive coping strategies [62,63]. Health and social care employers could make ‘stress and coping’ training mandatory as part of staff induction and annual refresher training/workshops to ensure a more resilient workforce. Intervention studies are needed, but the current results suggest that this training could help replace avoidance coping strategies with more adaptive ones, such as active coping and help-seeking. Second, we found that staff wellbeing was below the national average, which has implications for patient/service user care. Indeed, research has shown that poor staff wellbeing is associated with worse patient safety [33], and it can also lead to sickness absence, which has economic implications for the employer. Third, the fact that almost a third of our sample (31.17%) reported having low WRQOL should be a cause for concern. The UK health and social care system cannot afford to lose any part of its workforce, and employers should therefore listen to their employees, for example through staff feedback sessions [64] and support them in meaningful ways, especially during times of crisis, but also to build up coping skills and resilience. Other implications relate to work/life balance and how life outside work may contribute to stress or be helped with coping. The differences between respondents who were living alone and those who were not suggest new areas for employer support when normal social interactions outside the home may be limited.

## 5. Conclusions

The results from this survey provide the nursing, midwifery, AHP, social work, and social care workforce with a voice that evidences the impact of working during the first wave of the COVID-19 pandemic in the UK (May–July 2020). Importantly, this workforce intelligence provides baseline data for employers, policy makers, regulators, and professional bodies to better understand the wellbeing, coping, and WRQOL of the health and social care workforce during this period. The findings may be more applicable to the social work and social care workforce, which formed the majority of our sample, and may be less reflective of the job-related demands of healthcare workers in intensive environments (e.g., nurses in ICUs); however, they confirm expected results that wellbeing was significantly impacted. Additionally, they are also evidence that positive coping strategies are critical protective factors for the workforce.

## Figures and Tables

**Table 1:** Demographic information of the effective sample (*N* = 2541).

Variable	*n* (%)
Sex	
Male	320 (12.60)
Female	2211 (87.08)
Neither	8 (0.32)
Age	
16–29 years	305 (12.01)
30–49 years	1293 (50.93)
50–65 years	924 (36.39)
66+ years	17 (0.67)
Ethnicity	
White	2389 (94.17)
Mixed	46 (1.81)
Black	74 (2.92)
Asian	28 (1.10)
Relationship status	
Married	1337 (53.16)
Single	541 (21.51)
Divorced/separated	199 (7.91)
Cohabiting	438 (17.42)
Place of work	
England	905 (35.67)
Scotland	107 (4.22)
Wales	146 (5.75)
Northern Ireland	1379 (54.36)
Occupation	
Nursing	143 (5.63)
Midwifery	136 (5.35)
AHP	310 (12.20)
Social care	913 (35.93)
Social work	1039 (40.89)
Disability	
Yes	223 (8.78)
No	2260 (88.94)
Unsure	58 (2.28)
Number of sick days	
None	1196 (47.11)
Less than 10	973 (38.32)
11–20	158 (6.22)
21–60	136 (5.36)
More than 60	76 (2.99)
Redeployment	
Yes	359 (14.13)
No	2182 (85.87)

Note. Frequencies do not always add up to 2541 due to missing data. Presented are valid percentages.

**Table 2:** Final exploratory factor analysis results.

	Communality		Factor Loadings					
Item	Initial	Extracted	Active Coping	Avoidance Coping	Help Seeking	Religion	Substance Use	Humour
… trying to come up with a strategy about what to do	0.693	0.676	0.799					
… taking action to try to make the situation better	0.633	0.639	0.786					
… thinking hard about what steps to take	0.669	0.655	0.780					
… concentrating my efforts on doing something about the situation I’m in	0.565	0.523	0.705					
… trying to see it in a different light, to make it seem more positive	0.582	0.522	0.693					
… looking for something good in what is happening	0.578	0.509	0.647					
… accepting the reality of the fact that it has happened	0.558	0.379	0.528					
… learning to live with it	0.548	0.379	0.509					
… giving up the attempt to cope	0.550	0.424		0.624				
… giving up trying to deal with it	0.548	0.410		0.607				
… blaming myself for things that happened	0.486	0.379		0.583				
… criticizing myself	0.509	0.396		0.578				
… saying things to let my unpleasant feelings escape	0.376	0.358		0.556				
… refusing to believe that it has happened	0.421	0.302		0.535				
… saying to myself “this isn’t real”	0.376	0.230		0.463				
… expressing my negative feelings	0.317	0.257		0.428				
… getting help and advice from other people	0.682	0.697			0.796			
… getting emotional support from others	0.616	0.606			0.746			
… getting comfort and understanding from someone	0.620	0.612			0.742			
… trying to get advice or help from other people about what to do	0.644	0.578			0.704			
… praying or meditating	0.753	0.879				0.917		
… trying to find comfort in my religion or spiritual beliefs	0.753	0.793				0.867		
… using alcohol or other drugs to help me get through it	0.735	0.877					0.903	
… using alcohol or other drugs to make myself feel better	0.731	0.781					0.851	
… making jokes about it	0.628	0.818						0.887
… making fun of the situation	0.621	0.641						0.777

Note. Items ‘I’ve been turning to work or other activities to take my mind off things’ and ‘I’ve been doing something to think about it less, such as going to movies, watching TV, reading, daydreaming, sleeping, or shopping’ were omitted due to loadings of less than 0.30.

**Table 3:** Correlation matrix for continuous study variables (*N* = 2541).

	Variables												
Variables	1	2	3	4	5	6	7	8	9	10	11	12	13
1. Wellbeing	-	**0.548**	**0.371**	**−0.456**	**0.174**	**0.129**	**−0.226**	**0.071**	**0.091**	**0.212**	**0.263**	**0.252**	**0.213**
2. WRQOL	-	-	**0.215**	**−0.349**	**0.170**	**0.051**	**−0.143**	**0.067**	0.010	**0.159**	**0.238**	**0.246**	**0.161**
3. Brief COPE: Active coping	-	-	-	**−0.114**	**0.327**	**0.181**	**−0.104**	**0.150**	**0.155**	**0.160**	**0.340**	**0.216**	**0.165**
4. Brief COPE: Avoidance coping	-	-	-	-	**0.134**	**0.058**	**0.288**	**0.093**	**−0.075**	**−0.183**	**−0.115**	**−0.078**	**−0.110**
5. Brief COPE: Help seeking	-	-	-	-	-	**0.227**	0.011	**0.188**	**−0.080**	**−0.052**	**0.182**	**0.241**	**0.158**
6. Brief COPE: Religion	-	-	-	-	-	-	**−0.058**	0.001	0.009	0.034	**0.131**	**0.147**	**0.105**
7. Brief COPE: Substance use	-	-	-	-	-	-	-	**0.122**	**−0.081**	**−0.112**	**−0.117**	−0.018	**−0.041**
8. Brief COPE: Humor	-	-	-	-	-	-	-	-	**−0.051**	**−0.040**	0.031	**0.146**	**0.076**
9. Clark: Family-work segmentation	-	-	-	-	-	-	-	-	-	**0.494**	**0.175**	**0.049**	**0.072**
10. Clark: Work-family segmentation	-	-	-	-	-	-	-	-	-	-	**0.215**	**0.185**	**0.118**
11. Clark: Working to improve skills	-	-	-	-	-	-	-	-	-	-	-	**0.355**	**0.255**
12. Clark: Recreation and relaxation	-	-	-	-	-	-	-	-	-	-	-	-	**0.364**
13. Clark: Exercise	-	-	-	-	-	-	-	-	-	-	-	-	-

Note. WRQL = Work-related quality of life. Correlations based on Spearman’s rho. Significant correlations in bold (*p* < 0.05).

**Table 4:** Multiple regression estimates.

	Model 1: Wellbeing		Model 2: WRQOL	
Predictors	b ^1^	β ^2^	b ^1^	β ^2^
Coping strategies				
Brief COPE: Active coping	1.05	**0.19**	1.89	**0.08**
Brief COPE: Avoidance coping	−2.58	**−0.34**	−9.24	**−0.29**
Brief COPE: Help seeking	0.37	**0.08**	1.83	**0.09**
Brief COPE: Religion	0.18	**0.05**	0.32	0.02
Brief COPE: Substance use	−0.41	**−0.08**	−0.71	−0.03
Brief COPE: Humor	0.26	**0.07**	0.90	**0.06**
Clark: Family-work segmentation	−0.12	−0.03	−1.47	**−0.09**
Clark: Work-family segmentation	0.21	**0.06**	1.44	**0.10**
Clark: Working to improve skills	0.29	**0.09**	1.83	**0.13**
Clark: Recreation and relaxation	0.24	**0.08**	1.31	**0.10**
Clark: Exercise	0.13	**0.05**	−0.15	−0.01
Sex (ref. Female)				
Male	0.36	**0.03**	0.58	0.01
Age (ref. 16–29 years)				
30–49 years	0.06	0.01	−1.68	−0.06
50–65 years	0.59	**0.08**	−1.34	−0.04
66+ years	1.39	0.03	0.04	0.00
Ethnicity (ref. White)				
Mixed	0.07	0.00	−2.02	−0.02
Black	0.28	0.01	−4.04	**−0.04**
Asian	0.63	0.02	1.02	0.01
Relationship status (ref. Married)				
Single	−0.42	**−0.05**	−1.97	**−0.05**
Divorced/separated	−0.58	**−0.04**	−1.01	−0.02
Cohabiting	−0.19	−0.02	−2.38	**−0.06**
Place of work (ref. England)				
Scotland	−0.28	−0.02	−5.45	**−0.07**
Wales	0.17	0.01	1.12	0.02
Northern Ireland	0.39	**0.05**	−4.22	**−0.14**
Occupation (ref. Nursing)				
Midwifery	−0.07	−0.00	−1.91	−0.03
AHP	−0.72	**−0.07**	0.74	0.02
Social care	−0.36	−0.05	1.12	0.04
Social work	−0.57	**−0.08**	−0.38	−0.01
Disability (ref. No)				
Yes	−0.40	−0.03	−3.65	**−0.07**
Unsure	−0.60	−0.02	−3.80	**−0.04**
Number of sick days (ref. None)				
Less than 10	−0.38	**−0.05**	−0.98	−0.03
11–20	−0.68	**−0.05**	−2.92	**−0.05**
21–60	−0.54	**−0.03**	−3.22	**−0.05**
More than 60	−1.29	**−0.06**	−5.37	**−0.06**
Redeployment (ref. No)				
Yes	−0.20	−0.20	−2.45	**−0.06**

Note. ^1^ Unstandardized coefficient; ^2^ Standardized coefficient; WRQOL = Work-related quality of life; AHP = Allied Health Professional. Significant estimates in bold (*p* < 0.05).

## Data Availability

The data presented in this study are available on request from the corresponding author. The data are not publicly available due to ethical restrictions.
